# Is the priority review voucher program stimulating new drug development for tropical diseases?

**DOI:** 10.1371/journal.pntd.0006695

**Published:** 2018-08-09

**Authors:** Kirk W. Kerr, Thomas C. Henry, Kathleen L. Miller

**Affiliations:** 1 Office of Program and Strategic Analysis, Center for Drug Evaluation and Research, Food and Drug Administration, Silver Spring, MD United States of America; 2 Office of Planning, Office of the Commissioner, Food and Drug Administration, Silver Spring, MD United States of America; 3 Office of Orphan Products Development, Office of the Commissioner, Food and Drug Administration, Silver Spring, MD United States of America; 4 Department of Health Policy and Management, Gillings School of Global Public Health, University of North Carolina at Chapel Hill, Chapel Hill, NC United States of America; Northeastern University, UNITED STATES

## Abstract

Congress created the tropical disease priority review voucher program to stimulate new drug development for tropical diseases. An analysis of the pharmaceutical pipeline indicates that the development of drugs for these tropical diseases has increased. However, the effects of the program are not uniform across all diseases, as malaria and tuberculosis have seen significant new drug development, while other diseases have not.

## Author summary

Offering priority review vouchers (PRVs) as a reward for developing a drug to treat a tropical or other understudied disease was first introduced by Ridley et al (2006). The idea has become popular with lawmakers because it provides a perceived valuable incentive (vouchers have sold for as much as $350 million) without any additional appropriation of government funds. We investigate the effectiveness of these vouchers at stimulating drug development for tropical diseases. Using data from the PharmaProjects drug development database, we examine the number and development stage of drug development programs for voucher-eligible tropical diseases. We find that development for tropical diseases has increased, but that it has been concentrated in a few diseases with high incidence. We also note that the increase in development for tropical diseases coincides with increased development for all infectious diseases (of which tropical diseases are subset).

## Introduction

Prizes are one way that policymakers attempt to incentivize drug development in diseases that do not have satisfactory treatments. One such prize is the priority review voucher (PRV) program. [1] Created by Congress in 2007, the PRV program awards a voucher for the priority review of another drug to companies that develop a new drug for a tropical disease, as designated by Congress or the FDA. The program was later expanded to include both rare pediatric disease drugs, as well as medical counter-measure drugs.

When the PRV-eligible drug is approved by the US Food and Drug Administration (FDA), the company developing the drug receives a voucher for a priority review on another drug. These PRVs can be used by the company on one of their own drugs, or sold to another company.

Priority reviews are FDA reviews of new drug applications that have a goal completion time of six months, as opposed to the standard ten months. Typically, the priority review designation is given to drugs that treat serious diseases and provide a significant improvement over existing therapies. FDA prioritizes these drug reviews to enable faster patient access to important new therapies. A PRV allows drugs that would otherwise receive a standard review to receive the faster priority review.

As of July 2018, 22 PRVs have been awarded (six for tropical diseases (for malaria, tuberculosis, leishmaniosis, cholera, river blindness, and Chagas), fifteen for rare pediatric diseases, and one for a medical counter-measure). The first PRV was awarded in 2009. At least two companies have used the voucher for their own drugs, and the sale prices of eight PRVs have been publicly disclosed. The sale prices range from $67.5-$350 million, and the most recently disclosed sale price was $110 million.

Initial research, though inconclusive, suggests that the program has not increased new drug development in tropical diseases. [1, 2] Using a comprehensive drug development database, we find that development programs for voucher-eligible tropical diseases have increased since Congress enacted PRV legislation. However, the data contain additional nuances that make it difficult to fully attribute this increase to the PRV program.

## Background

The tropical disease PRV program covers twenty infectious diseases with differing incidences and standards of care. For example, tuberculosis affects millions of people worldwide and although effective pharmaceutical treatments exist for most infections, treatments for rarer, drug resistant infections are still needed. On the other hand, dracunuliasis affects fewer than 100 people each year and is preventable through basic public health measures, though no pharmaceutical intervention exists. S1 Appendix provides additional details on the incidence, prevention methods, and availability of treatments for PRV-eligible tropical diseases.

PRVs appear to be popular with legislators because they require no direct appropriation of federal funds; the voucher’s sources of value derive from coming to market earlier—increasing the time a drug is on the market before patent protection expires, and capturing market share, both of which are afforded by a faster FDA review. [3, 4] However, the program has raised multiple concerns, primarily centered on whether it is appropriate for a standard review drug to be reviewed in the shorter priority review period. [5, 6] Additionally, earlier research has demonstrated inconclusive results of the impact of vouchers on drug development for rare pediatric diseases. [1, 2]

In this analysis, we examine the impact of the PRV program, whether its impact differs by disease, and whether any impact differs from overall trends in the infectious disease drug space.

## Study data and methods

We collect data on drug development programs for tropical diseases from Citeline’s Pharmaprojects database. [7] The Pharmaprojects database compiles information on commercial drug development programs from public-domain sources as well as company contacts [Further information on the data can be found in S2 Appendix]. Pharmaprojects tracks drug development from the preclinical stage to worldwide market launch, and identifies programs that have been discontinued at any stage. This data affords us a more complete picture of drug development than examining the clinical trial stage alone.

We include in our dataset all tropical disease drug programs listed in Pharmaprojects as having at least some (preclinical or clinical) development. We exclude from our analysis the tropical diseases that were added to the PRV program between 2014 and 2016 (Chagas, Ebola, and Zika). Additionally, we identified the estimated incidence of the tropical diseases to provide for a sub-analysis of the data (S1 Appendix).

In addition to the data for the PRV-eligible diseases, we also compile data on drugs that Pharmaprojects classified under the disease category “infectious diseases” (exclusive of the tropical diseases studied here). We use this as a comparator group to assess any overall trends in drug development for this therapeutic area.

To evaluate the impact of the tropical disease PRV program, we compare the number of development programs starts before and after the PRV program was approved by Congress. If the program effectively incentivizes development for tropical diseases, we expect the number of development programs to increase. Since the overall number of drug development programs might be increasing regardless of the introduction of the PRV program, we compare the number of new tropical disease drug development programs with the number of development programs for infectious diseases generally.

While we rely primarily on graphical analysis for this paper, we also present a difference-in-difference regression model to support our findings (see S2 Appendix for a full description of regression methods).

## Study results

**Fig 1:**
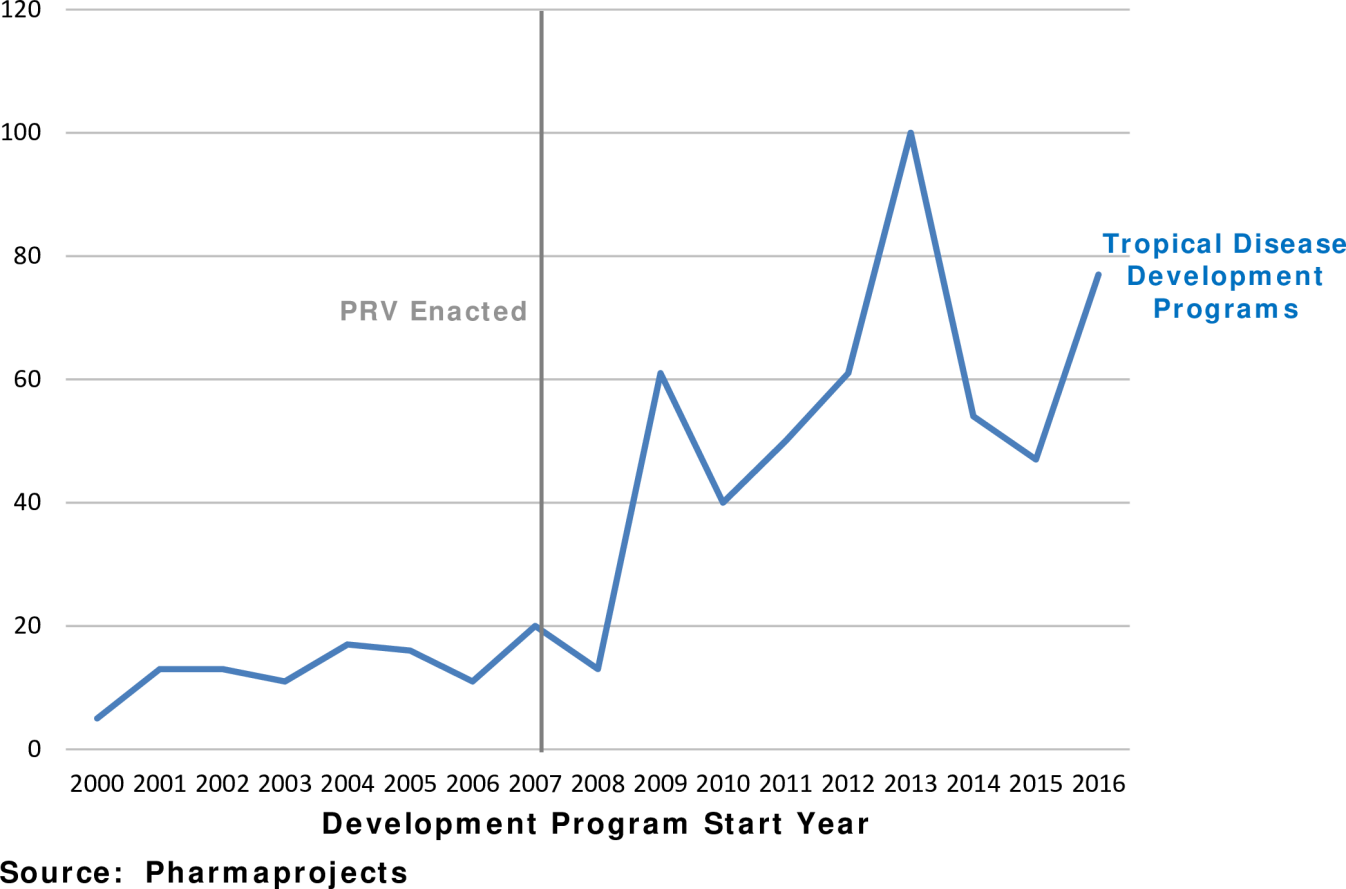
Drug development (preclinical and clinical) for tropical diseases has increased.

A total of 523 development programs have begun since the enactment of the PRV program. Fig 1 shows that drug development program starts have increased substantially since the PRV program began.

**Fig 2:**
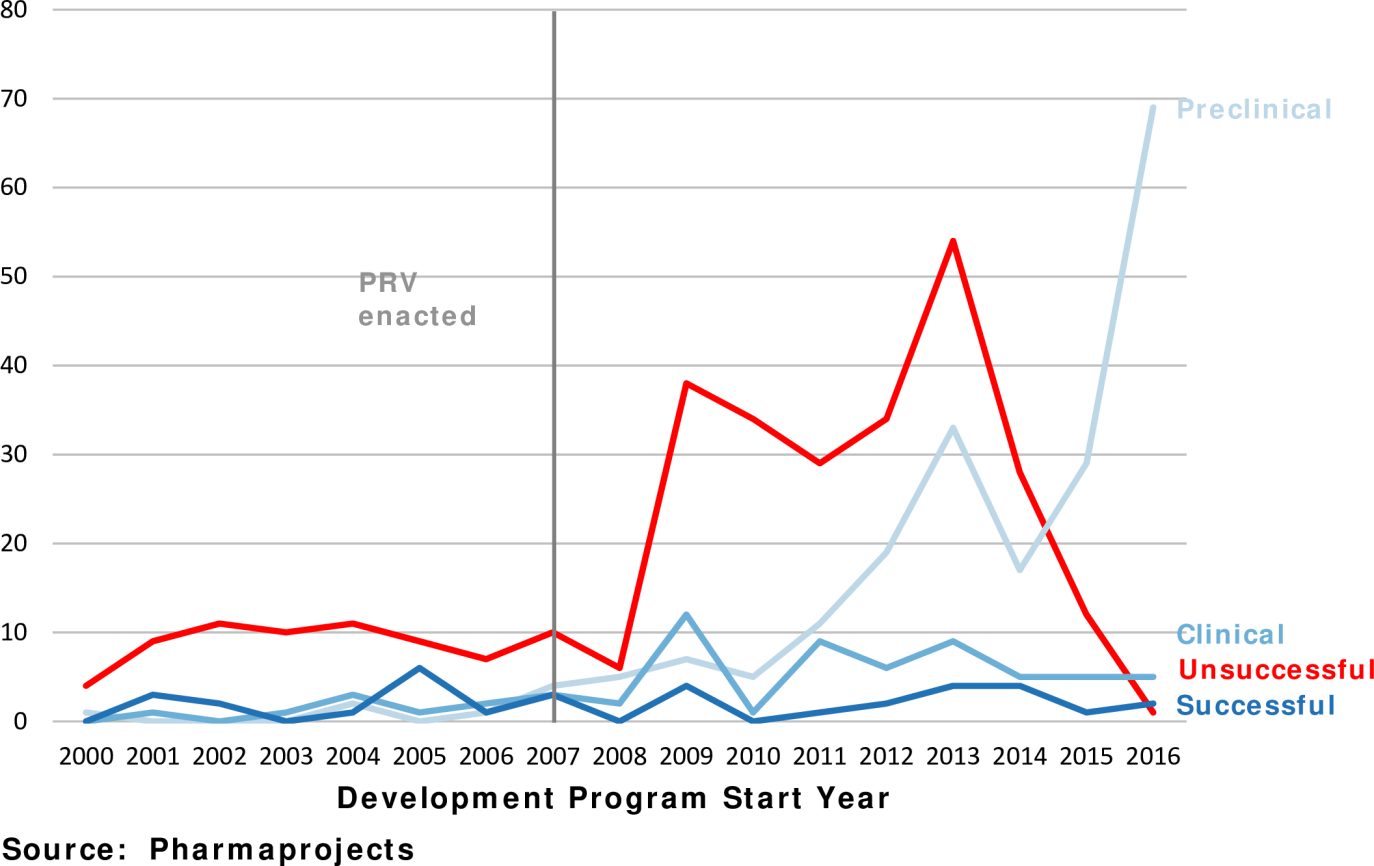
Most tropical disease development programs either unsuccessful or still in preclinical.

Fig 2 shows the current status of development programs for PRV-eligible diseases by the year the program began. For example, 38 development programs that began in 2009 ended unsuccessfully. Indeed, we find that 85% (445) of the programs have been either unsuccessful or are still in preclinical development.

**Fig 3:**
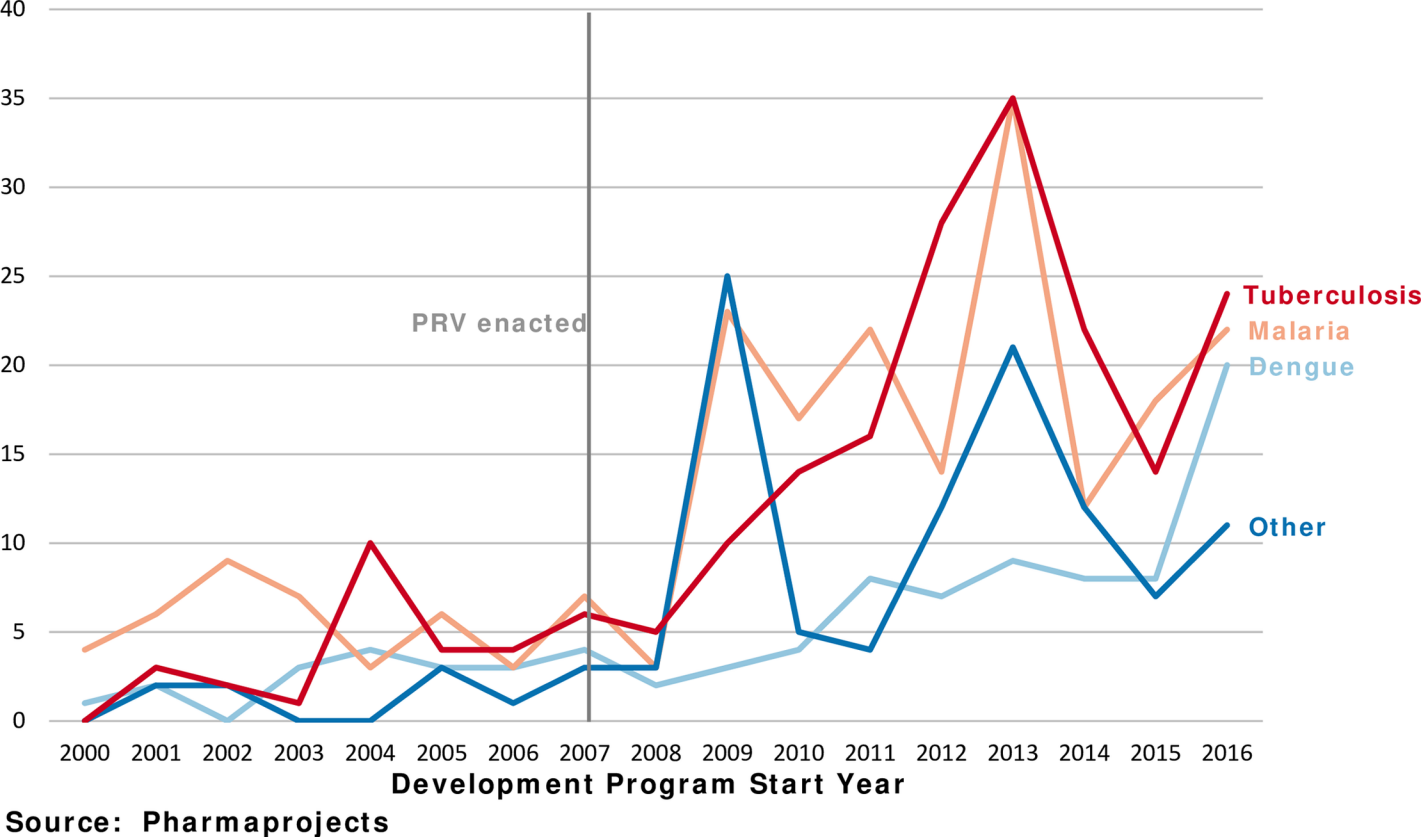
Notable growth for tuberculosis, malaria, and dengue.

We next consider the diseases for which voucher-eligible development takes place. Fig 3 shows the number of development programs begun for malaria, tuberculosis, dengue and a category that includes all the other PRV-eligible tropical diseases. Malaria and tuberculosis accounted for 66% (347) of development programs begun since 2007.

**Fig 4:**
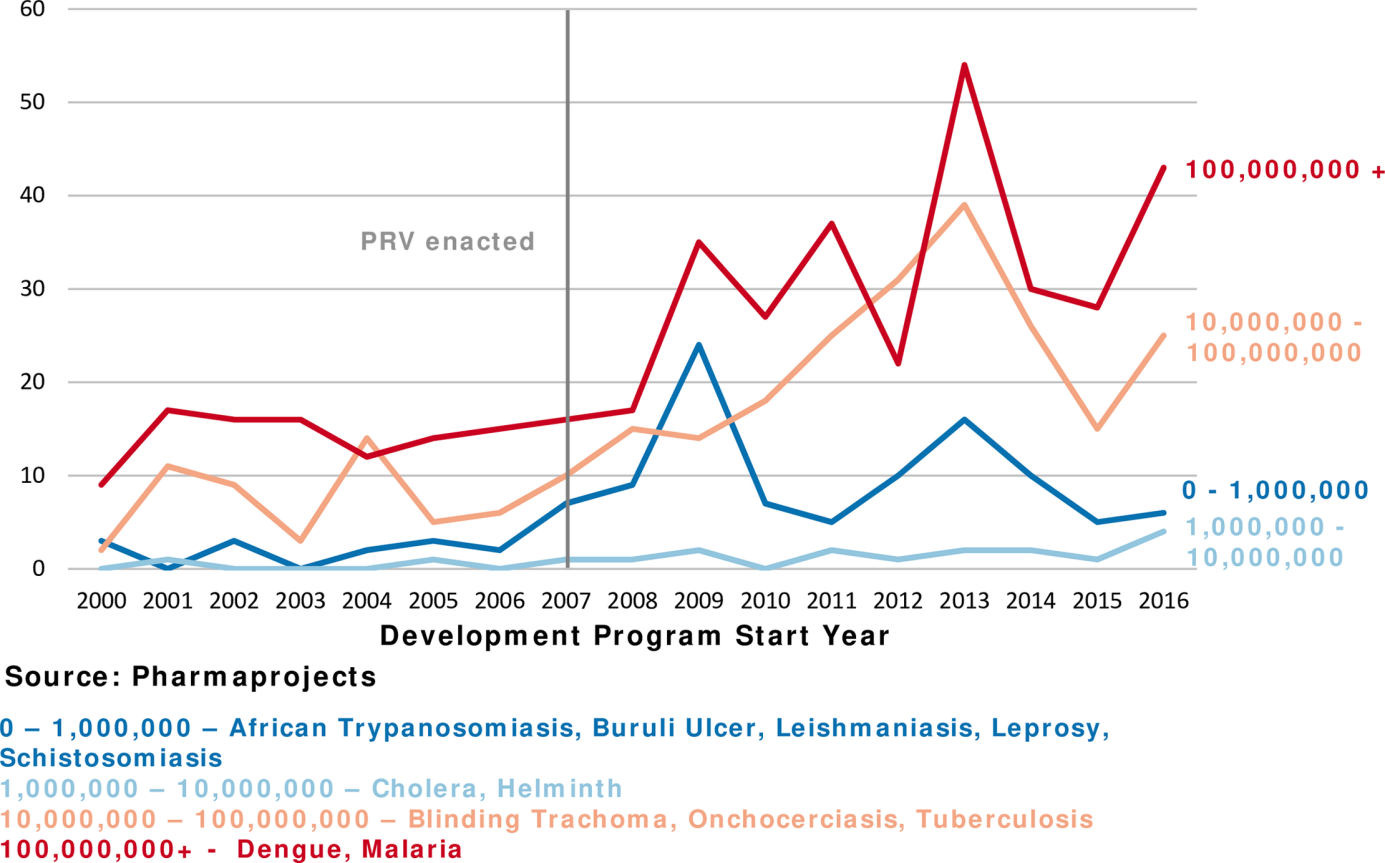
Most drug development (preclinical and clinical) in diseases with 10 million or more cases per year.

In Fig 4, we break out the number of drugs in development by the incidence of the disease being treated (see S1 Appendix for incidence data and sources). We find there is more development for diseases with higher incidence (greater than 10 million cases per year). In the most recent five years, 2012–2016, 85% of the new tropical disease development programs were for diseases which had incidence of at least 10 million people per year.

This proportion has also been increasing over time. An average of 21 new development programs were started each year for high-incidence diseases from 2000–2006. From 2007–2016, this average increased to 53 development programs per year, an increase of 152%. Development for low-incidence diseases also increased from an average of 2 new development programs per year to 12 new programs each year, giving it a much higher percentage increase (500%) even though the total number of programs is still low.

**Fig 5:**
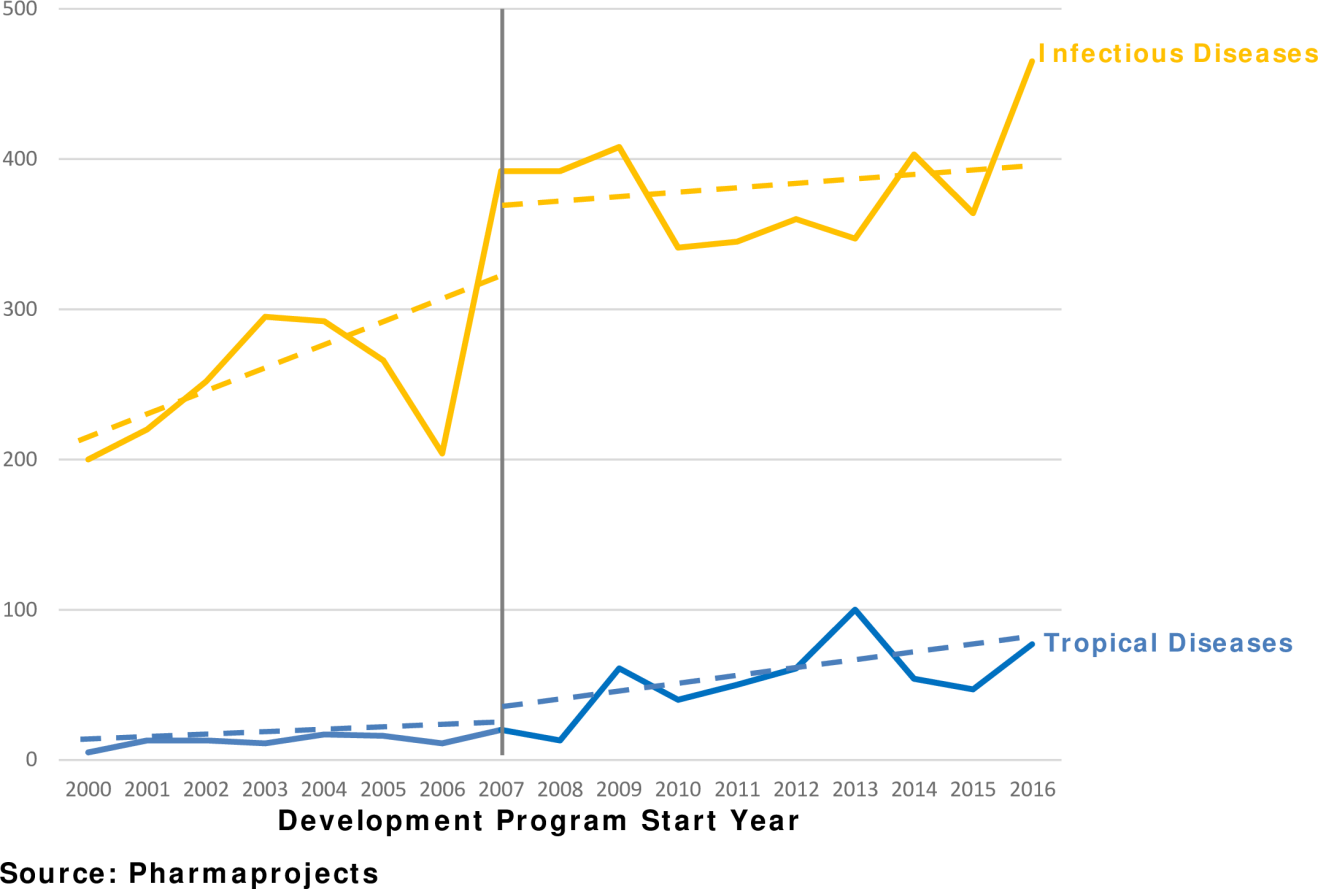
Infectious disease drug development (preclinical and clinical) increase more than tropical disease development.

Lastly, we compare tropical disease drug development to the development of drugs for infectious diseases. Fig 5 shows that the number of new infectious disease drug development programs increased in 2007 and continued at this approximate level. The trend in the number of new tropical disease drug development programs begun each year was increasing slightly over time before the PRV program was enacted. After the PRV program was enacted, the trend for new tropical disease programs increased, approaching overall trend for infectious disease programs.

To further support our graphical analysis of drug development, we performed a difference-in-difference regression (see S2 Appendix for full results). We found a positive, statistically significant result that indicates that the PRV program might have increased tropical disease drug development. The results indicated that the PRV program had a positive and statistically significant impact on the development of drugs for less common tropical diseases, but an only marginally significant impact on the number of malaria and tuberculosis development programs.

## Discussion

We find a significant increase in the development of new tropical disease drugs after Congress enacted PRV legislation, and this result was statistically significant in our regression analysis. Since most of the development programs commenced since the PRV program are still in very early stages (where ultimate approval rates are very low) or have already been abandoned, it is difficult to conclude definitively that the increase in drug development will lead to an increase in future approved therapies for these tropical diseases. Similarly, our ability to attribute this to the PRV program is limited because we see a similar trend in infectious disease drug development overall and we do not have complete confidence in the specification of our regression.

However, the trends that we see in new development for tropical diseases are correlated to PRV-related events. First, we see a large spike in development in 2009, corresponding to the year that the first PRV was awarded. It is possible that investors and developers needed to see that the FDA would award a voucher for a real increase in development to begin; there could also simply have been a lag between when the legislation was enacted to when companies first initiated development programs.

Second, we see a large increase in initial development in 2016, which is the year after multiple PRVs sold for hundreds of millions of dollars. Companies may have seen the high sale prices of the vouchers and decided to enter the tropical disease space.

There is also a large spike in development in 2013. However, we do not have a good rationale to explain it. It is likely that there are other external forces that are driving development in this space; for example, investment by the World Health Organization (that held an Assembly on tropical disease drug development in 2013) or nonprofit foundations, such as the Gates Foundation.

Additionally, our results indicate that drug development has increased for both high- and low- prevalence conditions. In the post-PRV period, development has centered on three diseases: dengue, malaria, and tuberculosis. This is not surprising, given that these are very high-prevalence conditions, which will therefore have large markets for approved drugs. However, the results of the statistical analysis indicate that only the increase in development of drugs for low-prevalence tropical diseases is statistically significant. Therefore, while the total number of development programs is larger for high-prevalence conditions, the proportional increase has been much higher for low-prevalence conditions as a group.

Lastly, an important conclusion of our results is that, while there have been many drug development programs begun for these diseases, the rate of failure remains high. Most tropical disease drug development programs begun between 2007–2013 have already failed, many without even reaching clinical development. This level of failure is not unusual in drug development. However, because the PRV is a prize awarded only after successful development and provides no compensation if development fails, it is possible that if failure rates remain high, companies will determine that the cost of development is not worth the potential prize, and drug development starts will return to pre-PRV levels.

Disclaimer: The views expressed in this article are those of the authors, and are not meant to represent the opinions of the Food and Drug Administration.

## Supporting information

S1 Appendix Tropical diseases–incidence, transmission, treatment and prevention. (PDF)

S2 Appendix Technical appendix. (PDF)

S3 Appendix Data. (XLSX)
