# Supplementary material for: Is the priority review voucher program stimulating new drug development for tropical diseases?
Source: PLoS Negl Trop Dis. 2018 Aug 9;12(8):e0006695. doi: 10.1371/journal.pntd.0006695 (PMC6103519; doi:10.1371/journal.pntd.0006695)
Supplement: S1 Appendix — (PDF) [file pntd.0006695.s001.pdf]

S1 Appendix

S1 Table 1: Tropical Diseases – Incidence, Transmission, Treatment and Prevention[i]

Disease	Estimated Incidence (year)	Transmission	Pharmaceutical Treatment Available	Vaccine Available	Non-medical Prevention and Treatment
African Trypanosomiasis	20,000 (2015)	Tsetse fly; contaminated body fluid	Yes	No	Vector control
Blinding Trachoma (Conjunctivitis)	85 Million (2016)	Contaminated body fluids; flies[ii]	Yes	No	Improved sanitation and hygiene; screening
Buruli Ulcer	2,037 (2015)	Unknown	Yes	No	Unknown
Chagas (American Trypanosomiasis)	6-7 Million (2016)	Triatomine bugs; contaminated body fluid; contaminated food	Yes	No	Vector control; improved hygiene, food preparation, and education
Cholera	1.3-4 Million (2015)	Contaminated water or food	Yes	Yes	Improved sanitation and food preparation
Dengue	284-528 Million (2013)[iii]	Aedes type mosquito; contaminated blood and organs	No	Yes	Vector control
Dracunculiasis (Guinea Worm Disease)	25 (2016)	Water fleas	No	No	Worm extraction; improved drinking water, education, and awareness
Ebola	28,616 (2014-2016)	Contaminated body fluids	No	No	Safe handling of infected animals and humans; surveillance and contact tracing
Fascioliasis (foodborne trematodiases)	2 Million (2013)[iv]	Contaminated fish, crustaceans or vegetables	Yes	No	Improved hygiene, food preparation, and sanitation
Filariasis (Lymphatic Filariasis – Elaphantiasis)	120 Million (2000)	Mosquitoes	Yes	No	Vector control; keep wound clean and move around if infected

Disease	Estimated Incidence (year)	Transmission	Pharmaceutical Treatment Available	Vaccine Available	Non-medical Prevention and Treatment
Leishmaniasis	700,000-1 Million (2016)	Female phlebotomine sandflies	Yes[e]	No	Vector control; disease surveillance
Leprosy (Hansen's Disease)	211,973 (2015)	Contaminated body fluid; armadillos	Yes	No	Avoid contact with rashes and bodily fluids of infected people
Malaria	212 Million (2015)	Female Anopheles mosquitoes	Yes	Yes	Vector control
Marburg	2 (2008)	Rousettus bat colonies; contaminated body fluid	No	No	Avoid contact with infected humans and animals; prompt and safe burial
Onchocerciasis (River Blindness)	18 Million (2016)	Blackflies	Yes	No	Vector control and avoidance
Schistosomiasis (bilharzia)	66.5 Million (2015)	Contaminated water	Yes	No	Vector control; improved hygiene and sanitation; safer water
Soil Transmitted Helminthiasis	1.5 Million (2016)	Contaminated vegetables, water, or soil	Yes	No	Improved hygiene, sanitation, and health education
Tuberculosis (TB)	10.4 Million (2015)	Contaminated body fluid	Yes	Yes	Health education
Yaws	46,000 (2015)	Contact with infected wound	Yes	No	Health education; improved personal hygiene
Zika	440,000-1.3 Million (2015)[v]	Aedes mosquitoes; sexual transmission	No	No	Vector control; rest, hydration, and pain treatment if infected

[1] Source: http://www.who.int, unless otherwise noted
[2] Source: http://emedicine.medscape.com
[3] Bhatt, S. et. al, "The Global Distribution and Burden of Dengue," Nature, 2013 April 25; 496(7446):504-7.

[4] Source: http://www.cdc.gov

[5] Only Brazil; Source: European Centre for Disease Prevention and Control, Stockholm. Zika virus disease epidemic: potential association with microcephaly and Guillain-Barré syndrome (first update). Available at http://ecdc.europa.eu/en/publications/Publications/rapid-risk-assessment-zika-virus-first-update-jan-2016.pdf, January 21, 2016
