# Supplementary material for: Is the priority review voucher program stimulating new drug development for tropical diseases?
Source: PLoS Negl Trop Dis. 2018 Aug 9;12(8):e0006695. doi: 10.1371/journal.pntd.0006695 (PMC6103519; doi:10.1371/journal.pntd.0006695)
Supplement: S2 Appendix — (PDF) [file pntd.0006695.s002.pdf]

S2 Appendix: Technical Appendix

The Pharmaprojects database compiles information on commercial drug development programs from public-domain sources (company press releases, and web pages, Company analyst presentations and annual reports, primary news sources, and journals via PubMed,) as well as from company contacts.[i] Citeline claims that Pharmprojects "covers the progress of all significant new drug candidates as they enter commercial pharmaceutical research and development programmes around the world."[ii] The data tracks drug development as early as preclinical development up to market launch worldwide and also identifies programs that have been discontinued at any stage. We identify drug development program starts by the earliest event listed in PharmaProjects. We define a drug development program as discontinued when PharmaProjects identifies it as discontinued or determines that no development has taken place in the past 12 months.

We supplement our descriptive analysis with a basic difference-in-differences regression. The difference-in-differences method compares outcomes for control and treatment groups over time, using the timing of treatment application to identify the treatment's impact. In this case, PRV-eligible tropical disease drugs are the treatment group and other infectious diseases are the control group. In addition to a treatment group that includes all PRV-eligible tropical disease drugs, we create a treatment group for malaria and tuberculosis drugs and a second treatment group for all other PRV-eligible drug development programs. Treatment application occurs in the period after enactment of the PRV legislation (i.e. post 2007). The effect of the PRV legislation is measured by the interaction of the treatment group with the treatment period. Our unit of observation is the number of development programs begun each year for the different disease groups (i.e. tropical vs. non-tropical or malaria and tuberculosis, other tropical, and non-tropical diseases).

We present the results of our regression in table A2.1. Regressions using all PRV-eligible tropical disease drugs as a single treatment group are shown in columns 1 and 2. The interaction of the treatment group and treatment period (Tropical Disease – PRV interaction) is positive and statistically significant in specifications with and without year fixed effects. Columns 3 and 4 present results using the split treatment groups of malaria and tuberculosis, and all other PRV-eligible tropical diseases. Again, the interaction of the treatment period with the treatment group(s) is positive and statistically significant for both the malaria and tuberculosis treatment group and the other tropical diseases treatment group.

S2 Table 1

Regression on Log Number of Drug Development Program Starts

	(1)	(2)	(3)	(4)
Tropical Disease	-2.965***	-2.965***		
	0.2	0.189		
Malaria & Tuberculosis			-3.258***	-3.258***
			0.229	0.171
Other Tropical Diseases			-4.080***	-4.080***
			0.229	0.171
PRV Enacted	0.441**	1.270***	0.441**	1.393***
	0.184	0.375	0.211	0.29
Tropical Disease - PRV Interaction	0.865***	0.865***		
	0.261	0.247		
Malaria & Tuberculosis - PRV Interaction			0.757**	0.757***
			0.298	0.222
Other Tropical Diseases - PRV Interaction			0.919***	0.919***
			0.298	0.222
Constant	5.502***	5.030***	5.502***	4.981***
	0.141	0.268	0.162	0.209
Year Fixed Effects	No	Yes	No	Yes
Observations	34	34	51	51
R-squared	0.934	0.97	0.942	0.978

Standard errors in parentheses

*** p<0.01, ** p<0.05, *p<0.1

These regressions have several limitations. First, our regressions are limited to only a few possible determinants of drug development program starts. There are likely other determinants for which we do not have data, such as potential revenue and scientific advances, and are unable to include. Also, our sample size is quite small due to the limited amount of time since PRV enactment. Finally, the drug development trends for tropical diseases and infectious diseases are not parallel. Non-parallel treated and control groups make the comparison of tropical disease and infectious disease drug development trends before and after enactment of the PRV less informative. Bearing these limitations in mind, our regressions support our general conclusion that the PRV sparked an increase in tropical disease drug development.

---

[1] https://fda-pipeline.citeline.com/CpHelp.aspx accessed Aug. 24, 2017

[2] Pharmaprojects Scope Statement
